# Segrosome Complex Formation during DNA Trafficking in Bacterial Cell Division

**DOI:** 10.3389/fmolb.2016.00051

**Published:** 2016-09-09

**Authors:** María A. Oliva

**Affiliations:** Department of Chemical and Physical Biology, Centro de Investigaciones Biológicas, Consejo Superior de Investigaciones CientíficasMadrid, Spain

**Keywords:** DNA segregation, partitioning systems, segrosome, partitioning complex, nucleoprotein complex, ParB, ParR, TubR

## Abstract

Bacterial extrachromosomal DNAs often contribute to virulence in pathogenic organisms or facilitate adaptation to particular environments. The transmission of genetic information from one generation to the next requires sufficient partitioning of DNA molecules to ensure that at least one copy reaches each side of the division plane and is inherited by the daughter cells. Segregation of the bacterial chromosome occurs during or after replication and probably involves a strategy in which several protein complexes participate to modify the folding pattern and distribution first of the origin domain and then of the rest of the chromosome. Low-copy number plasmids rely on specialized partitioning systems, which in some cases use a mechanism that show striking similarity to eukaryotic DNA segregation. Overall, there have been multiple systems implicated in the dynamic transport of DNA cargo to a new cellular position during the cell cycle but most seem to share a common initial DNA partitioning step, involving the formation of a nucleoprotein complex called the segrosome. The particular features and complex topologies of individual segrosomes depend on both the nature of the DNA binding protein involved and on the recognized centromeric DNA sequence, both of which vary across systems. The combination of *in vivo* and *in vitro* approaches, with structural biology has significantly furthered our understanding of the mechanisms underlying DNA trafficking in bacteria. Here, I discuss recent advances and the molecular details of the DNA segregation machinery, focusing on the formation of the segrosome complex.

## DNA maintenance during bacterial cell division

The process of DNA segregation is a crucial stage of the bacterial cell cycle and it depends on the precise coordination with other cellular events. The faithful inheritance of genetic information during cell division ensures that each daughter cell receives a copy of the newly replicated DNA. In many organisms, the DNA-encoded genome consists of a core genome (the chromosome) and accessory genomes (extra-chromosomal, mobile genetic elements, MGEs). MGEs (plasmids, phages, conjugative transposons, etc.) often confer evolutionary advantages to the host bacteria, including the adaptation to different environmental niches. Many, if not most, naturally occurring MGEs are in low or unique copy number and thus bring their own post-replication survival apparatus encoded in stability determinants (partitioning systems, toxin-antitoxin systems, and multimer-resolution systems).

Partitioning (*par*) systems help to reliably segregate sister DNAs via a process that could be seen as functionally analogous to the mitotic segregation of chromosomes in eukaryotic cells. The best studied and probably the most common partitioning systems constitute a compact genetic module that is tightly auto-regulated by one of the gene products and consists of only three elements: a cis-acting DNA sequence and two trans-acting proteins. The DNA sequence denotes a *par* site or centromere-like region, and can be located at a single site (upstream or downstream of the operon) or at multiple positions within the MGE. The trans-acting proteins consist of a centromere-binding protein (CBP) that binds to the centromere and forms a nucleoprotein complex (partition complex or segrosome), and a motor protein (an NTPase), that sometimes is a cytomotive filament, which effectively moves the MGE inside the bacteria through direct interaction with the segrosome. Initially, these systems were classified as follows, based on the molecular nature of the NTPase: type I (Walker A-type ATPase), which further divided into Ia and Ib based on differences in the trans-acting proteins and the position of the centromere in the operon; and type II (cytomotive, actin-like ATPase) (Gerdes et al., 2000). Recently, an additional type III system has emerged, in which a cytomotive, tubulin-like GTPase serves as the motor protein (Larsen et al., 2007). Further, there may exist a type IV partitioning system, in which only a cis-acting DNA site and a DNA binding protein seem required for plasmid maintenance (Simpson et al., 2003; Guynet and de la Cruz, 2011). Hence, they may use a host bacteria's motor protein to track the DNA, or may even segregate passively by establishing an association with the chromosome (Guynet and de la Cruz, 2011).

It seems that partitioning systems share a common initial step that involves the specific recognition of the centromeric DNA region by the CBP. This step is crucial for assembly of the segrosome and subsequent events during DNA segregation. However, there is a considerable divergence among *par* sites and CBPs display different domain folds and organization (Hayes and Barilla, 2006; Baxter and Funnell, 2014), indicating differences in the segrosome assembly process and by extension, the corresponding partitioning mechanism. Here, I review the molecular mechanisms underlying segrosome formation in the partitioning systems that have been studied, focusing on those where structural information is available. Despite these variations in centromere sequences and the natures of the CBPs, common to all systems is the formation of the nucleoprotein complex that I propose may be categorized into two classes: those that mediate DNA segregation via *bridging* and those that do so via *wrapping*.

## DNA bridging in type Ia partitioning systems

Many plasmids, phages and chromosomes encode type Ia partitioning systems (Martin et al., 1987; Balzer et al., 1992; Lewis and Errington, 1997; Grigoriev and Lobocka, 2001). No single, common segrosome assembly mechanism has been described for these systems, probably owing to the wide diversity of centromeres and variations on the CBPs (below). The exact nature of the partition complex is unknown, if it even exists in only one particular conformation, but the CBP bridges distant regions of DNA via both specific and non-specific binding (Rodionov et al., 1999; Bingle et al., 2005; Murray et al., 2006; Schumacher et al., 2007b; Graham et al., 2014), enabling the formation of a nucleoprotein complex linking and/or spanning thousands of base pairs with a small number of CBPs. Furthermore, spreading of the CBP has a masking effect on the function of the covered DNA, preventing interaction between the motor protein and the DNA and favoring the interaction with the segrosome (Bouet et al., 2007).

While the sequences of cis-acting sites (*parS, sopC* or O_B_) vary, the sites always contain inverted repeats. The *parS* site contains two different repeats asymmetrically arranged around a binding site for the IHF protein (Davis and Austin, 1988; Funnell, 1988b). One of the motifs is a heptamer (A-box) and the other a hexamer (B-box). Binding of IHF bends the DNA by 180°, thus strongly promoting ParB binding (Funnell, 1988a; Funnell and Gagnier, 1993; Rice et al., 1996; Bouet et al., 2000; Surtees and Funnell, 2001). Some chromosomes contain several *parS* sites dispersed over ~15% of the DNA, surrounding the replication origin (Lin and Grossman, 1998; Livny et al., 2007). However, the chromosomal *parS* sites consist exclusively of palindromic A-box motifs. *SopC* and O_B_ sites comprise only one type of short inverted repeats contained within longer iterons that can be found either at a single locus (Mori et al., 1986) or scattered across the genome (Balzer et al., 1992; Ravin and Lane, 1999). The function of the regions flanking the inverted repeats is puzzling, as their presence is not conserved (Ravin and Lane, 1999). Similarly, the need for more than one iteron remains unclear, as in almost all cases a single copy is sufficient for segregation (Martin et al., 1987; Williams et al., 1998; Yates et al., 1999). However, given that the full-length centromere maximizes partitioning efficiency (Martin et al., 1987), the architecture of each segrosome may reflect evolutionary pressure on how well the systems work.

**Figure 1:**
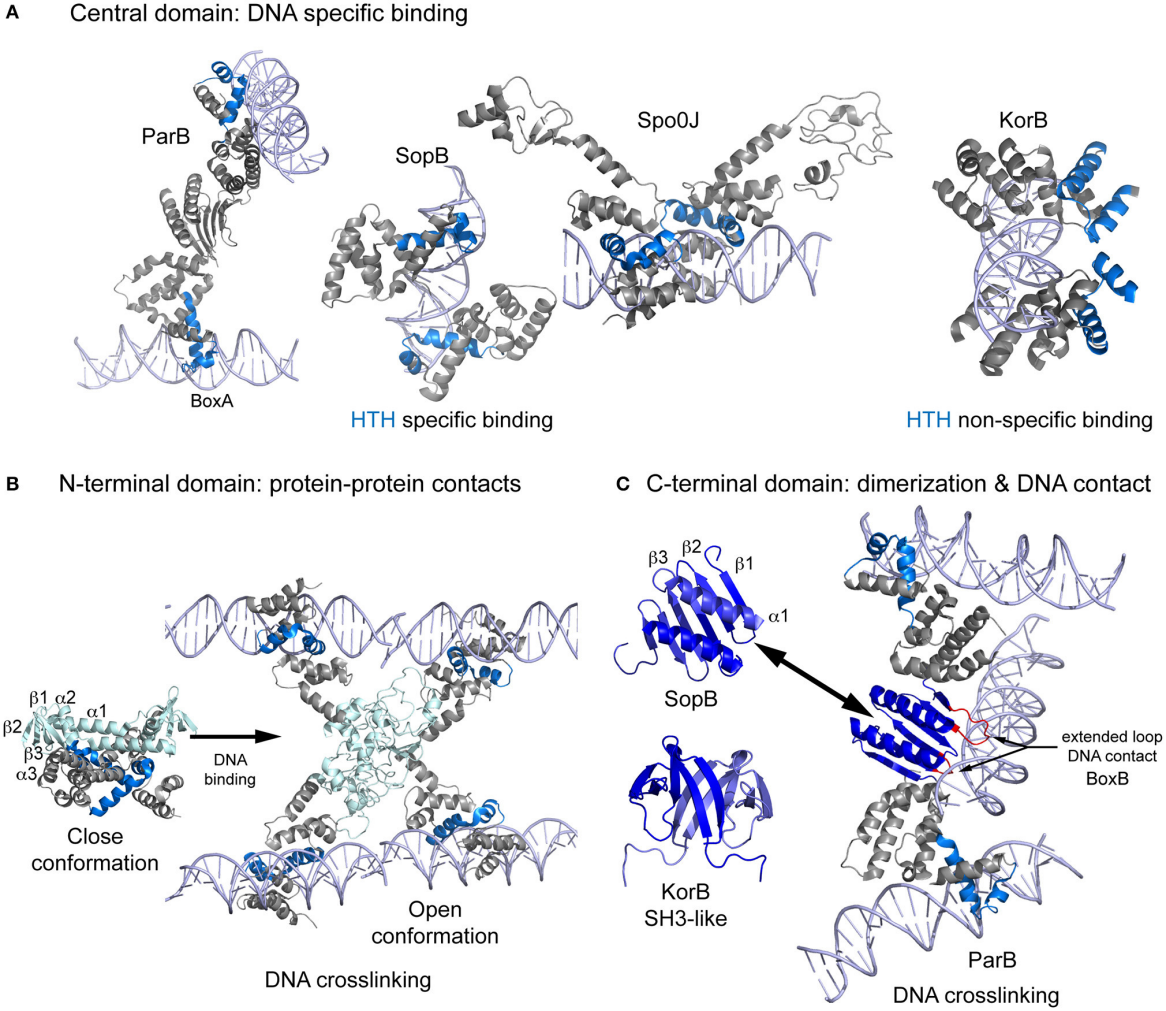
**Scheme showing the structures of Type Ia CBPs domains and their interaction with DNA during bridging. (A)** Central domain showing primary specific DNA interaction of ParB, SopB, Spo0J, and KorB. These proteins bind as dimers, making contact with both sides of the DNA. However, ParB dimerization generate a different contact mechanism that involves the bridging of different DNA molecules. **(B)** Spo0J N-terminal domain structures, both unbound and DNA-bound. Binding to the centromere induces a domain rearrangement that favors DNA bridging. **(C)** The C-terminal domain folding differs considerably between ParB/sopB and KorB. ParB folding includes extended loops that make contacts with DNA, favoring bridging distant molecules. DNA is shown in light purple, the HTH motif in blue, the N-terminal domain in light blue, and the C-terminal domain in dark blue.

Type I CBPs are members of the ParB protein superfamily but show low sequence conservation. ParB, Spo0J, SopB, and KorB share the same domain organization, consisting of three flexibly linked domains (Schumacher et al., 2010): N-terminal, central (with a DNA-binding helix-turn-helix, HTH, motif), and C-terminal domains, which have been seen in various inter-domain conformations (Chen et al., 2015). The central domain is responsible for the primary CBP-DNA interaction, and the N- and C-terminal domains contribute to CBPs spreading around the centromere DNA. ParB proteins show high structural conservation only in the central domain, probably due to the presence of the HTH motif. For DNA binding, the HTH recognition helix inserts into the major groove, but there are differences between CBPs. In ParB, the HTH motif binds the *parS* box-A exclusively via the recognition helix (Schumacher and Funnell, 2005). SopB uses the recognition helix and an Arg outside the HTH (Schumacher et al., 2010; Sanchez et al., 2013). Spo0J binding is very similar to that observed for SopB but uses a Lys instead of an Arg and form additional specific contacts via another Arg and a Glu (Chen et al., 2015). Surprisingly, the HTH motif of KorB mediates only non-specific interactions, and DNA binding specificity depends on contacts formed via a Thr and an Arg located outside the HTH (Khare et al., 2004). All these proteins bind DNA as dimers, whereby each molecule generally interacts with opposite sides of the inverted repeat (Figure 1A). However, in the crystal structure of ParB, the monomers of each dimer bind to box elements of different DNA molecules, suggesting a possible DNA crosslinking function or, that crystal packing occluded correct binding (Schumacher and Funnell, 2005).

ParBs' flexible N-terminal domain is responsible for the binding to the motor protein, oligomerization of the CBP around the centromere, and also loading of bacterial condensin (Gruber and Errington, 2009; Sullivan et al., 2009; Minnen et al., 2011; Havey et al., 2012; Graham et al., 2014). Figure 1B shows the domain topology of Spo0J (α1-β1-β2-α2-β3-α3), in which β-strands fold to form a β-sheet (Leonard et al., 2004). The two conserved motifs, box 1 and box 2 [with an “arginine patch,” (Yamaichi and Niki, 2000)] are located between α1 and β1 and between β2 and α2, respectively (Chen et al., 2015). Upon DNA binding the protein opens into an elongated, 78Å long structure, leaving the N-terminal domain exposed and available for protein-protein interactions (Chen et al., 2015). These interactions are very flexible, but always include box 1 and 2 (Kusiak et al., 2011). Through this arrangement, the N-terminal domain is able to assist CBP spreading (Kusiak et al., 2011; Graham et al., 2014). Due to a lack of structural data, it remains unclear how the flexibility of the domains and their binding to DNA enables simultaneous or alternative interactions with the condensin and motor proteins.

The C-terminal domain is the most divergent, but in all these proteins shares the ability to dimerize (Leonard et al., 2004; Chen et al., 2015). The domain topology of ParB/SopB is β1-β2-β3-α1, where the β3 s of each monomer combine to form a continuous 6-stranded β-sheet and the helices interact to form an antiparallel coiled-coil (Figure 1C, Schumacher and Funnell, 2005; Schumacher et al., 2010). ParB contains extended loops between β1-β2 and β2-β3 that form highly specific contacts with the *parS* B-box (Schumacher and Funnell, 2005), generating a secondary DNA binding domain and contributing to DNA bridging during segrosome formation. By contrast, the C-terminal domain of KorB displays a completely different folding pattern, resembling an SH3 protein and consisting of a 5-stranded antiparallel β sheet (Delbruck et al., 2002). However, crosslinking studies suggest that this domain also facilitates DNA binding (Delbruck et al., 2002).

## Segrosome assembly via wrapping

This strategy involves the formation of a filamentous nucleoprotein complex, in which the CBP wraps the centromere (type Ia partition systems) or the DNA wraps around a CBP oligomer (type II and III partition systems). The resulting segrosome is a single and discrete structure.

### Type Ib systems

**Figure 2:**
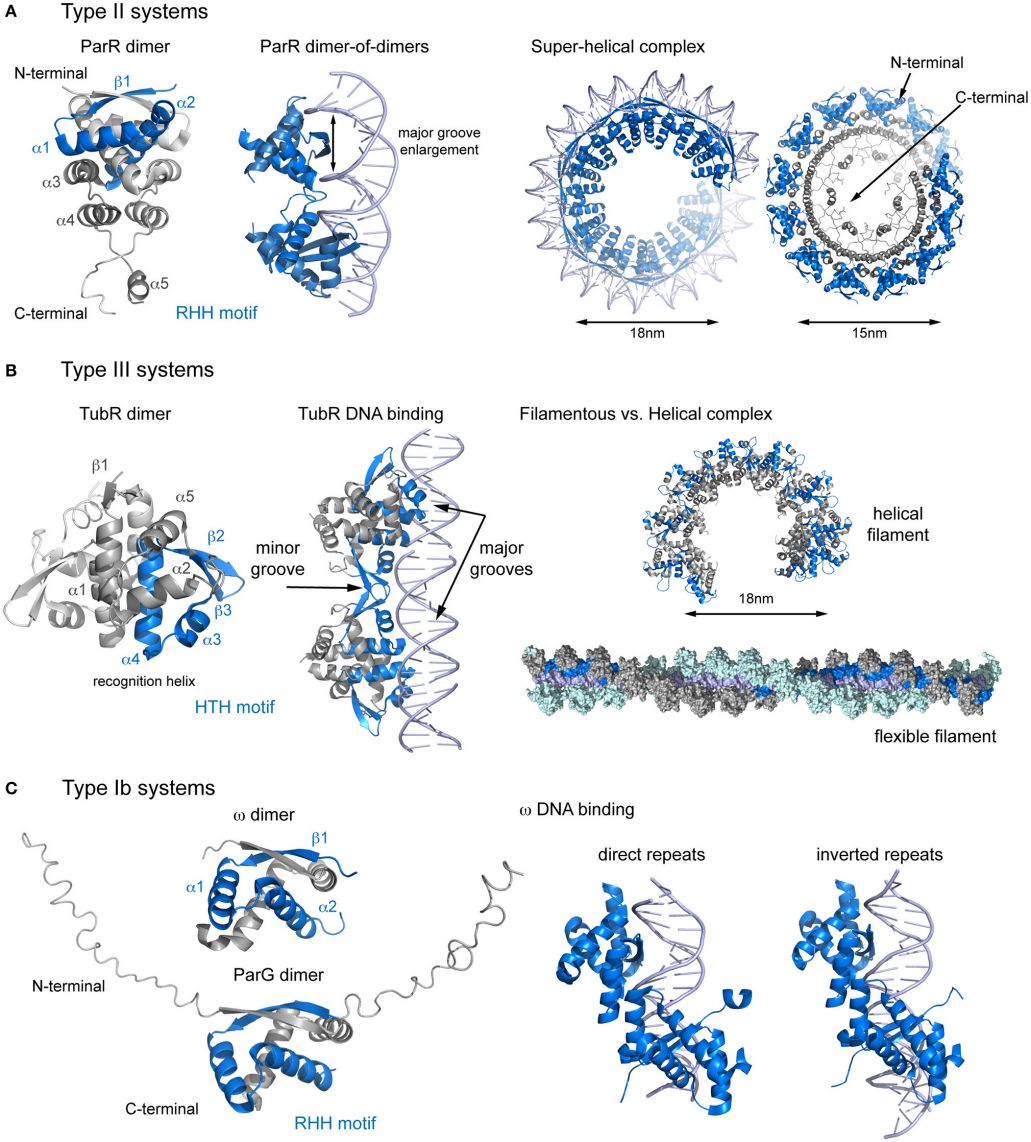
**Structural comparison of CBPs involved in segrosome assembly by wrapping**. **(A)** Type II partition systems. Structure of ParR dimer (left), showing topology and the RHH motif; DNA changes upon ParR binding (middle), with enlargement of the DNA major groove; and formation of the the segrosome complex by DNA wrapping of the ParR super-helical oligomer (right), leaving the ParR C-terminal tail in the helix inside. **(B)** Type III partition systems. Structure of TubR dimer (left), showing topology and the HTH motif; the TubR-DNA binding mechanism (middle), in which the HTH makes contacts with the DNA major groove and the wing forms contacts with the minor groove; and putative filamentous vs. helical segrosome complexes (right), according to two crystal packing arrangements. **(C)** Type Ib partition systems. Structures of ParG and ω dimers (left), showing the RHH motif and the flexible N-terminal domain, and protein binding to direct and inverted repeats in equivalent ways (right).

Surprisingly, the arrangement of the components in Type Ib systems is the only common aspect shared with the aforementioned systems. The interactions between their main components are different, and so may be the segregation mechanism. The centromere site localizes upstream of the *par* operon and consists of direct and inverted repeats. However, in plasmid pCXC100 the centromeric site contains only direct repeats (Yin et al., 2006; Huang et al., 2011). The CBPs, which also functions as repressors (Carmelo et al., 2005; Weihofen et al., 2006) are small proteins that share the arrangement into N- and C-terminal domains (Figure 2C). The N-terminal domain, which shows a highly divergent sequence, is flexible and unstructured, and includes a conserved arginine finger that has been implicated in the activation of ATP hydrolysis in the motor protein (Barilla et al., 2007). The C-terminal domain topology is β1-α1-α2 and includes a ribbon-helix-helix (RHH) DNA-binding motif (Murayama et al., 2001; Golovanov et al., 2003; Huang et al., 2011). The β1 strand from two different molecules pairs into an antiparallel β-ribbon, meaning that these CBPs are also present as dimers in solution (Barilla and Hayes, 2003; Golovanov et al., 2003).

Plasmid pSM19035 harbors a unique partitioning system: rather than being encoded in a single operon, each gene is transcribed separately from different promoters. The full centromere contains 3 separate *parS* sites, consisting of 9, 7, and 10 iterons that occur twice in the plasmid genome (*parS1, parS1*′, *parS2, parS2*′, *parS3, parS3*′, de la Hoz et al., 2000, 2004; Dmowski et al., 2006). However, *parS2* appears to be the main centromeric sequence (Dmowski and Kern-Zdanowicz, 2016). Interestingly, each *parS* overlaps with the promoters of genes involved in plasmid copy number and maintenance: *parS1* with Pδ, *parS2* with Pω and *parS3* with P_copS_ (de la Hoz et al., 2000). The CBP, ω, binds to each *parS* with different affinities, depending on the number of iterons (de la Hoz et al., 2004). This feature may be crucial to fine-tune repressor affinity for different promoters (Weihofen et al., 2006). The nucleoprotein complex is a left-handed protein helix that wraps the DNA (Weihofen et al., 2006) covering only the *parS* site (Pratto et al., 2009). Protein binding to both direct and inverted repeats involves comparable interactions, due to the pseudo-symmetry of the dimer (Weihofen et al., 2006, Figure 2C). Binding induces minor structural changes mainly affecting the loop connecting α1 and α2. In contrast to other RHH DNA-binding proteins, there is no DNA bending (Pratto et al., 2009). Because the DNA is not curved, ω first makes contact with the DNA major groove via base specific interactions with residues on the β-sheet and then the N-termini of the α2 helices clamp the phosphate backbones (Weihofen et al., 2006). Assuming nearly straight DNA, the number and orientation of repetitions will affect the distances between helices α1 of adjacent ω dimers, thereby modulating the cooperativity. The motor protein, δ, binds non-specifically to DNA but is recruited to the location of the segrosome (Pratto et al., 2009) to form a ternary complex, giving rise to intermolecular pairing of *parS* regions (Pratto et al., 2008, 2009). This bridging may increase the local concentration of ω, in turn increasing the ATPase activity of the motor protein and thus inducing detachment of this protein and promoting mobility (Pratto et al., 2009). This system may combines both DNA wrapping mechanisms (during segrosome formation) and DNA bridging mechanisms (when the motor protein participates in segregation).

In plasmid TP228, the centromere (*parH*) is continuous and consists of direct and inverted repeats separated by AT-rich regions. A DNA region between the operon genes and the centromere (O_F_) comprises more repeats that play important roles in partitioning and transcription regulation (Zampini et al., 2009; Wu et al., 2011). Binding of the CBP, ParG, to *parH* occurs via the RHH motif, but unlike in ω, ParG is also dependent on the protein's N-terminal tail, which modulates binding affinity (Golovanov et al., 2003). Apparently, the AT-enriched spacers may increase the binding cooperativity of ParG to DNA (Wu et al., 2011). Like plasmid pSM19035, the centromere site is not curved and ParG binding does not induce DNA bending. The motor protein, ParF polymerizes into filaments and does not bind to DNA (Barilla et al., 2005; Schumacher et al., 2012). The N-terminal domain of ParG is not only important for the activation of the ATPase but also facilitates ParF filament nucleation and bundling (Barilla et al., 2007). Furthermore, in contrast to all other described systems, the ParF-ParG interaction is not dependent on the formation of the segrosome. This suggests that pSM19035 and TP228 despite sharing the same type Ib partitioning system employ distinct segregation mechanisms.

### Type II systems

The centromeric site (*parC*) consists of tandem repeats localized in a single locus upstream of the operon. The arrangement can be continuous (plasmid pSK41, Schumacher et al., 2007a) or split into two regions (plasmid R1), with the par cassette promoter in the middle (Dam and Gerdes, 1994). However, the resulting segregation complexes are very similar. The CBP, ParR, contains two domains, an N-terminal domain with a RHH DNA binding motif (as seen in type Ib systems), and a C-terminal domain that is involved in the interaction with the motor protein. The domain topology of the ParR N-terminal domain is β1-α1-α2-α3-α4-α5 (Figure 2A). The β1-strands from two monomers combine in an antiparallel fashion and the α1-α2 helices come together to form an extensive dimer (Moller-Jensen et al., 2007; Schumacher et al., 2007a). The C-terminal domain includes a 3-helix cap that reinforces the tight dimerization of the N-terminal domain and an unstructured C-terminal tail with a high degree of sequence conservation (Moller-Jensen et al., 2007).

The nucleoprotein complex forms a discrete helical arrangement with a diameter of 15-nm (Moller-Jensen et al., 2007; Hoischen et al., 2008). The structure of the nucleoprotein complex (Schumacher et al., 2007a) reveals a continuous helical array in the crystal packing (Figure 2A). Each turn consists of 6 symmetrical pairs of dimers (involving the assembly of 12 ParR dimers), producing distinct negative and positive electrostatic on the inner and outer surfaces of the helix. The DNA wraps ParR by interacting with the outer, positively charged surface of the super helix, with each dimer binding one *parC* iteron. When the centromere is split in two, the promoter region forms a DNA loop that protrudes out of the ParR-*parC* ring structure (Hoischen et al., 2008; Salje and Lowe, 2008), repressing the promoter (Jensen et al., 1994; Breuner et al., 1996), and regulating transcription of the partition genes (Salje and Lowe, 2008). The DNA is bent by 46° and widened so that the major groove grows from 11 to 14Å (Schumacher et al., 2007a). The groove enlargement allows the insertion of the RHH motif, as described for other DNA-binding RHH proteins (Somers and Phillips, 1992; Raumann et al., 1994; Gomis-Ruth et al., 1998). Interestingly, the phosphate contacts cluster at the 5′ ends of the 10-bp repeats, creating the closest physical associations between ParR and the DNA. Full-length ParR from plasmid pB171 crystallized in a helical superstructure in the absence of DNA, with a diameter very similar to that measured in the nucleoprotein complex (15 vs. 18 nm) (Figure 2A). Moreover, the protein arrangement into dimers and the electrostatic distribution are also similar (Moller-Jensen et al., 2007). These observations lead to the question; which event occurs first? If ParR assembly into a super-helical structure occurs first, then the macromolecular complex may recruit *parC*. Otherwise, the centromere might function as a scaffold for ParR oligomerization.

For ParR, the segrosome structure positions the conserved C-tails clustered on the inside surface of the helix, where they mediate binding to the motor protein, ParM (Schumacher et al., 2007a; Salje and Lowe, 2008). The ParR tail binds to a hydrophobic pocket in ParM in an interaction resembling that described for actin polymer modulators and the barbed end of actin filaments (Gayathri et al., 2012). Furthermore, the segrosome binds only at the growing end of the polar ParM double helical filament favoring filament growth via a formin-like mechanism (Gayathri et al., 2012). Why does the ParR-ParM interaction require the clustering of so many ParR tails? It is possible that several tails bind to a single ParM molecule with distinct affinities, regulating ParM filament dynamics. Alternatively, the presence of free tails may be necessary to explore the space around the filament end and to facilitate the addition of ParM molecules to the growing filament while remaining attached at all times.

### Type III systems

The type III system were the most recently discovered partitioning systems (Larsen et al., 2007). For TubZRC, the centromeric site (*tubC*) is localized upstream of the operon and contains several direct repeats in a single locus that can be split into two (pBtoxis) or three (pBsph) blocks, resembling discontinuous *parC* sites (Aylett and Lowe, 2012; Ge et al., 2014a). During partitioning, the CBP (TubR in this case) mediates the assembly of the segrosome nucleoprotein complex and acts as a repressor of *tubRZ* transcription (Tang et al., 2006; Larsen et al., 2007; Ge et al., 2014a). TubR is a small winged-helix DNA-binding protein with a high degree of structure conservation. The topology is β1-α1-α2-α3-α4-β2-β3-α5, where the α3-α4 helices form the HTH motif (α4 is the “recognition helix”) and the loop between β2-β3 forms the wing (Figure 2B, Ni et al., 2010). Interestingly, TubR forms a highly intertwined dimer involving the canonical HTH motif, resulting in an atypical protein-DNA binding (Aylett and Lowe, 2012). The N-termini of both recognition helices in a dimer protrude into the major groove of the DNA, while the acidic patch in the wing complements the DNA backbone phosphate in the minor groove. The nucleoprotein complex takes the shape of a flexible filament, with TubR wrapping helically around both sides of *tubC* (Aylett and Lowe, 2012, Figure 2B). The filamentous complex closes to form 18-nm wide ring-like structures (Aylett and Lowe, 2012). However, the structure of plasmid pBM400 TubR, with no DNA bound, reveals a helical arrangement, resembling the ParR super-helical complex (Figures 2A,B). It thus remains unclear whether the segrosome complex is formed by TubR wrapping of the DNA or by DNA wrapping of the TubR oligomer, which could lead to different interacting mechanism with the motor protein (TubZ).

TubR binds to TubZ C-terminal tail (Ni et al., 2010). However, the interaction is only possible following formation of the filamentous segrosome. Neither TubR alone (Oliva et al., 2012) nor TubR bound to either of the two-iteron clusters are capable of interacting with TubZ (Aylett and Lowe, 2012; Fink and Lowe, 2015). Therefore, the clustering of TubR may generate the binding site for TubZ. Differently to type II systems, the segrosome tracks the shrinking minus end of the TubZ filament, suggesting a pulling segregation mechanism (Fink and Lowe, 2015).

Type III partition systems involve a third protein with a predicted HTH DNA-binding motif and a long coiled-coil domain (TubY), located downstream of the partition operon (Oliva et al., 2012). TubY seems to be a regulator protein that modulates TubZ assembly (Oliva et al., 2012) and also acts as a transcriptional activator (Ge et al., 2014b) but the exact molecular mechanisms remain elusive.

It is still common for new partitioning systems to be discovered in plasmids, phages, and on chromosomes. Together with a growing body of molecular insights these will help to broaden our understanding of DNA trafficking during bacterial cell division and in particular how DNA is attached to the CBP during segrosome formation and then to the motor protein through the segrosome.

## Author contributions

MO conceived and wrote this mini-review.

### Conflict of interest statement

The author declares that the research was conducted in the absence of any commercial or financial relationships that could be construed as a potential conflict of interest. The handling Editor declared a shared affiliation, though no other collaboration, with the author and states that the process nevertheless met the standards of a fair and objective review.
